# Adherent Pericardium in Children

**Published:** 1894-06

**Authors:** Theodore Fisher

**Affiliations:** Registrar to the Bristol General Hospital


### ADHERENT PERICARDIUM IN CHILDREN.

BY

Theodore Fisher, M.D. Lond.,

*Registrar to the Bristol General Hospital.*

---

I wish to bring forward a few facts in connection with adherent pericardium in children, the two most important of which are—(1) that adherent pericardium is the most common cause of enlarged heart in children, and (2) that this sequence of pericarditis is much more serious in its nature than valvular disease, and that children in whom it is present will probably never reach adult life. These facts are not new; but although known to those who have had experience of children, they do not appear to have received general recognition. The two writers who most clearly give expression upon this point are Dr. Sansom and Dr. Sturges, both of whom are physicians to children's as well as to general hospitals.

Dr. Sansom, comparing the effect of pericarditis in children and in adults, makes the following statement: "Pericarditis in childhood may pass away leaving no trace, but more frequently it is accompanied by persisting endocardial changes, and is followed by adhesions between the visceral and parietal layers of the pericardium with much thickening—a condition which, however it may be in the adult, disposes the child to very considerable hypertrophy of the heart."[1] He also says: "In children adhesions of the pericardium constitute one of the most important conditions that militate against recovery. A valvular lesion may be perfectly compensated, but pericardial adhesions are adverse, and often lead to rapid death."[2] Here we have adherent pericardium referred to both as a cause of hypertrophy of the heart and of rapid death.

Dr. Sturges lays stress upon the same points. When speaking of hypertrophy of the heart, he says: "Of the origin

[1] *The Diagnosis of Diseases of the Heart and Thoracic Aorta*, 1892, p. 38.

[2] *Ibid*, p. 105.

of this change it does not become us to speak positively in the patient's lifetime. But we know for certain, and can show you in our Museum, that the common cause is adherent pericardium."[1] Dr. Sturges emphasises the importance of pericarditis as a complication of endocarditis. He mentions mitral stenosis as the most common valvular lesion, but says: "The well-being of a child having this defect depends largely on his immunity from subsequent rheumatic attacks, however slight; and what chiefly abridges the period of health he will enjoy is the occurrence of adherent pericardium either in the first or in some later attack."

During a recent visit to London, I examined the *post-mortem* reports of Guy's Hospital for the last seven years, in order to see what evidence they afforded on these points. The following table shows clearly the association of enlarged heart with general adhesion of the pericardium. It must be remembered that the normal weight of the heart at the age of four is about 2 oz., between four and seven years 2⅔ oz., and between seven and fourteen years 4⅓ oz.

| Sex. | Age. | Weight of Heart. | Condition of Valves. |
|---|---|---|---|
| M. | 3½ | 7½ oz. (with pericardium) | Normal. |
| M. | 7 | 7 oz. ... ... | Small bead-like vegetations on mitral valves; valves otherwise normal. |
| M. | 8 | 10 ,, ... ... | Small bead-like vegetations on mitral and aortic valves; valves otherwise normal. |
| M. | 9 | 7½ ,, ... ... | Valves normal. |
| F. | 9 | 12 ,, ... ... | Mitral valves thickened. |
| M. | 13 | 15 ,, ... ... | Valves normal. |
| F. | 11 | 17 ,, ... ... | Mitral valves thickened. |
| M. | 12 | 13 ,, ... ... | Valves normal. |
| F. | 12 | 8½ ,, ... ... | Valves normal. |
| F. | 12 | 23 ,, ... ... | Mitral valves slightly thickened; small bead-like vegetations on aortic valves. |
| M. | 13 | 22 ,, ... ... | Valves normal. |
| F. | 15 | 13½ ,, ... ... | Small bead-like vegetations on aortic and mitral valves; valves otherwise normal. |
| F. | 15 | 12 ,, ... ... | Mitral valves thickened and shrunken; aortic valves also thickened, but "not shrunk." |

[1] *Lancet*, 1892, vol. i., p. 622.

In the above cases adherent pericardium may be said to have been the cause of death, and the associated valvular disease was a comparatively unimportant complication. In nine out of the thirteen cases the valves were distinctly stated not to have been thickened, and in the report of the remaining four it is stated only once that the valves were shrunken.

That adherent pericardium, apart from any valvular disease, may be a cause of great enlargement is seen from the case, in a boy aged 13, in which the heart weighed 22 oz., while the valves were quite normal. In one other case, in which the heart of a girl weighed 23 oz., it is distinctly stated in the report of the necropsy that although the valves were thickened, the condition of the orifice itself "would have caused no regurgitation." In three of the cases in which the valves were not thickened, small bead-like vegetations were present. These vegetations were, however, too small to have had an injurious effect upon the circulation through the heart, and cannot prevent us from considering the adhesion of the pericardium as the cause of the enlargement.

Not only is adherent pericardium a serious form of heart-disease in children, but it appears to be more common than ordinary valvular disease. In the records for the seven years from which the above cases were taken there were only four cases of death from valvular disease in children aged fifteen years and under, and one of those four gave evidence of previous pericarditis. In a fifth case, myocarditis was thought to have been the cause of death, and here also there was evidence of a former attack of pericarditis. Excluding this last case, we have four cases of death from valvular disease and thirteen of death from adherent pericardium. From so small a list it perhaps would hardly be safe to draw any sweeping conclusion, but the above ratio is to a great extent corroborated by Dr. Sansom,[1] who says that out of ninety-nine cases that showed clinical evidence of mitral regurgitation no less than three-fourths gave *post-mortem* evidence of pericarditis.

The other point to which I wish especially to refer is the

[1] *Cyclopædia of Diseases of Children*, Ed. by Keating, 1890, vol. ii., part ii., p. 821.

prognosis, which appears to be more serious in these cases than in valvular disease. A child with adherent pericardium will if it lives to puberty, probably not survive that period. The time from seven to fourteen years of age is one of the most active periods of the growth of the heart, and while the circulatory disturbance produced by valvular lesions may be efficiently combated by healthy hypertrophy, the presence of adherent pericardium will probably lead to a final breakdown. Between the ages of fifteen and twenty the Guy's Hospital *post-mortem* records for the same period of seven years give four cases only in which adherent pericardium was present, and in three of these there was also serious valve-disease. Above the age of twenty there was no instance in which adherent pericardium could have been said to have been the cause of death, since the associated valve-disease was well-marked. From the above facts we may infer that below the age of fifteen adherent pericardium is the most serious form of cardiac disease, between fifteen and twenty that it is less commonly fatal, and that above the age of twenty it very rarely causes death. To put this conclusion into other words, we may say that adherent pericardium is the form of chronic heart-disease most to be feared in children, while in adults valvular disease is undoubtedly of far greater consequence.

Adherent pericardium in children being of such serious nature, the importance of diagnosis is at once obvious. This is a somewhat difficult question. If aware of a previous attack of pericarditis in a child suffering from an enlarged heart, we should probably diagnose adherent pericardium on the grounds of probability, but in the absence of such a history we are driven to rely upon physical signs alone. Here a text-book list arises before us: retraction of the apex-beat, retraction of the lower end of the sternum, diastolic rebound, pulsation of the veins of the neck, the pulsus paradoxus, and so on. I may say, perhaps with little fear of contradiction, that all these are of little value. To take the first, the retraction of the apex-beat, for example; it is generally considered not to occur unless there are adhesions between the pericardium, the pleura, and the chest-wall as well as between the two surfaces of the pericardium. These adhesions are, however, the exception rather than the rule;[1] so that, allowing the retraction to be present in every case in which they exist, the sign would in the majority of instances fail us. But when such adhesions are present the retraction may be absent. In a case that recently died under the care of Dr. Harrison in the Bristol General Hospital, I suspected adherent pericardium during life, and examined the cardiac impulse very carefully. At the *post-mortem* examination the pericardium was found to be universally and firmly adherent to the chest wall, yet clinically there had been nothing but the forcible slapping impulse of a dilated heart, which had something of that undulating character not uncommonly seen. Again, allowing retraction of the apex-beat to be present in some cases of adherent pericardium, it may also exist where there is no suspicion of cardiac disease; and thus its value is still further diminished. The clinical reports of the cases dying in Guy's Hospital are silent upon this point and upon retraction of the lower end of the sternum. This does not prove that the signs were in every case absent; but since the reports are written under careful supervision, it is obvious that if present they were not sufficiently well-marked to attract attention.[2] While retraction of the apex-beat and of the lower end of the sternum are not mentioned, "a curious recession" of the impulse was noticed during diastole in one case. Although this is the reverse of diastolic rebound, there must have been something abnormal occurring after the impulse. Signs such as pulsation of the veins in the neck and the pulsus paradoxus need not detain us; they are still less distinctive.

[1] Dr. Wordsworth Poole, who has recently examined the reports of cases of adherent pericardium at all ages dying in Guy's Hospital during ten years, kindly tells me that adhesions between the heart and the chest-wall occurred only 11 times in 196 cases.

[2] Since this paper was written I have examined the *post-mortem* records of cases of enlargement of the heart dying in Guy's Hospital during the space of twenty years, and cases of adherent pericardium in children during twenty-seven years. These reports were examined with the view of elucidating another question, and special attention was not given to the points referred to above. Three instances, were, however noted in which retraction of the apex-beat, or of the chest-wall in its immediate neighbourhood, had been present. The importance of adherent pericardium in children was well illustrated; but in one or two isolated instances it seemed to be the main cause of death in patients of nearly thirty years of age. These, however, were exceptions; between twenty and thirty, deaths from valvular disease, most commonly mitral stenosis, were greatly in the majority.

Reference may, however, be made to a physical sign upon which some writers lay considerable stress. That is, that the situation of the apex-beat does not alter with the position of the patient. The impulse of a healthy heart shifts ½ in. to ¾ in. or more when the patient turns from lying on the back to the left side; but when adherent pericardium is present, it is said to remain stationary. It is obvious, however, that adhesion must also be present between the pericardium and the chest-wall; and since these are generally absent, this sign can be of no great value. In two cases in which I have had good reason to suspect adherent pericardium, since a dilated heart has followed a history of pericarditis, I have found that the impulse altered its position as in the hearts of healthy children.

It is to an auscultatory sign that I wish particularly to refer. That sign is a sound heard during diastole which may constitute part of a typical *bruit de galop*, or be a rumbling sound that might easily be mistaken for a presystolic bruit. In cases of adherent pericardium with dilated heart, whether there is disease of the mitral valves or not, we shall almost certainly have a systolic apex bruit due to regurgitation of the heart. In addition to this systolic bruit, we shall also probably hear another abnormal sound interpolated between the first and second sounds. This may be merely like a soft first or second sound, or it may be of rumbling character, diastolic or presystolic in time, and thus be thought to indicate mitral stenosis. The two varieties were well illustrated in the case of adherent pericardium under Dr. Harrison already referred to. When first examined, a presystolic rumbling sound, followed by the first sound, and a systolic bruit were audible at the apex, but six days after the presystolic rumble had become diastolic and formed part of a *bruit de galop*. That the presystolic rumbling sound was not due to any mitral stenosis is shown by the fact that at the *post-mortem* examination the orifice admitted five fingers. As before mentioned, this bruit, audible in the dilated heart of a case of adherent pericardium, may be either diastolic or presystolic. The clinical reports of the thirteen cases that occurred in Guy's Hospital showed that a presystolic rumbling sound was noted twice and a diastolic bruit three times. The distinction of these sounds from those due to mitral stenosis must sometimes be very difficult. The presytolic rumble of a dilated heart is, however, very low pitched, and the disastolic bruit will probably be heard not only just round the impulse, as is common in mitral stenosis, but also over the right ventricle in the third and fourth intercostal spaces.[1]

I must only briefly touch upon the pathology of the enlargement of the heart. That it is not due to mere adhesion of the pericardium is obvious, since the heart does not enlarge when the adhesion follows left-sided pleurisy, or occurs in patients dying of phthisis.[2]

The rheumatic pericarditis of children must have peculiarly virulent qualities not possessed by pericarditis due to local causes. That it commonly proves fatal in the acute stage shows that the heart may be profoundly affected at the onset. One naturally thinks of the heart-muscle, but microscopic examination does not always reveal any marked change; for example, I have sections of heart-muscle in my possession, from a case of pericarditis in a girl aged fourteen, that show little, if any, alteration in the muscle-fibres; in fact, nothing abnormal but a few leucocytes surrounding the blood-vessels close under the pericardium. Perhaps in these cases the nerve-supply to the heart suffers. But whatever the explanation may be of the serious results of pericarditis in children, the fact remains that those results are serious, and far more serious than has been generally recognised, since valvular disease still occupies the foremost place in works upon diseases of children, adherent pericardium being often disposed of in one or two sentences.

[1] The occurrence of a presystolic or diastolic apex murmur apart from stenosis of the mitral orifice and of disease of the aortic valves appears to have escaped notice until this year. Since this paper was written, Dr. Sturges has mentioned in the Lumleian Lectures five instances of a presystolic murmur without stenosis in children, and Dr. Graham Steell has reported in the *Practitioner* for April two cases in which a diastolic murmur occurred in the dilated hearts of patients with adherent pericardium. The ages of these patients were 19 and 23.

[2] Dr. Wordsworth Poole, who was examining the Guy's Hospital *post-mortem* reports of adherent pericardium at the same time as myself, tells me that during ten years there were eight cases of adherent pericardium associated with old left-sided pleurisy or with phthisis in children under the age of fifteen, and in only one was the heart enlarged.

